# Association of sleep duration and snack consumption in children and adolescents: The CASPIAN‐V study

**DOI:** 10.1002/fsn3.1471

**Published:** 2020-02-19

**Authors:** Nafiseh Mozaffarian, Ramin Heshmat, Asal Ataie‐Jafari, Mohammad Esmaeil Motlagh, Hasan Ziaodini, Gita Shafiee, Majzoubeh Taheri, Morteza Mansourian, Mostafa Qorbani, Roya Kelishadi

**Affiliations:** ^1^ Department of Pediatrics, Child Growth and Development Research Center, Research Institute for Primordial Prevention of Non‐communicable Disease Isfahan University of Medical Sciences Isfahan Iran; ^2^ Chronic Diseases Research Center, Endocrinology and Metabolism Population Sciences Institute Tehran University of Medical Sciences Tehran Iran; ^3^ Department of Nutrition, Science and Research Branch Islamic Azad University Tehran Iran; ^4^ Department of Pediatrics Ahvaz Jundishapur University of Medical Sciences Ahvaz Iran; ^5^ Ministry of Education Tehran Iran; ^6^ Office of Adolescents and School Health Ministry of Health and Medical Education Tehran Iran; ^7^ Health Management and Economics Research Center Iran University of Medical Sciences Tehran Iran; ^8^ Non-communicable Diseases Research Center Alborz University of Medical Sciences Karaj Iran; ^9^ Endocrinology and Metabolism Research Center, Endocrinology and Metabolism Clinical Sciences Institute Tehran University of Medical Sciences Tehran Iran

**Keywords:** children and adolescents, food habits, sleep duration

## Abstract

**Objectives:**

The relationship between sleep deprivation and the risk of overweight and obesity is somewhat known in children and adolescents. This study aimed to investigate the relationship between sleep duration and eating snacks in a national sample of children and adolescents aged 6–18 years old.

**Methods:**

This cross‐sectional study was carried out on the data of the fifth survey of the national school‐based surveillance system entitled the “Childhood and Adolescence Surveillance and PreventIon of Adult Non‐communicable Disease” (CASPIAN‐V) study. Short sleeping duration was defined 10 hr per day for children under 10 years and 9 hr per day for children ≥ 10 years. To assess food habits, the consumption frequency of some food groups including sweets, salty snacks, carbonated beverages, diet soft drinks, soft beer, fresh fruits, dried fruits, fresh juices, vegetables, packed juices, dairy products (milk, yogurt, and cheese), fast foods, tea, sugar along with tea, and coffee was asked using Likert scale ( never, rarely, weekly, and daily).

**Results:**

In multivariate model, short sleep versus long sleep in students was associated with increased chance of eating salty snacks (OR = 1.49 [95% CI: 1.38–1.61]; *p* = .001), soft drinks (OR = 1.12 [95% CI: 1.04–1.20]; *p* = .002), fast foods (OR = 1.66 [95% CI: 1.54–1.79]; *p* < .001), tea (OR = 1.49 [95% CI: 1.39–1.61]; *p* < .001), and tea with sugar (OR = 1.13 [95% CI: 1.05–1.22]; *p* < .001). In addition, short sleep in students was associated with a decreased odds of daily intake of soft drinks without sugars (OR = 0.64 [95% CI: 0.58–0.70]; *p* < .001), soft beer (OR = 0.92 [95% CI: 0.85–0.99]; *p* < .001), fresh fruit (OR = 0.83 [95% CI: 0.76–0.90]; *p* < .001), dried fruit (OR = 0.43 [95% CI: 0.39–0.46]; *p* < .001), fresh fruit juice (OR = 0.66 [95% CI: 0.62–0.72]; *p* < .001), packed juice (OR = 0.91 [95% CI: 0.84–0.98]; *p* < .009), milk (OR = 0.51 [95% CI: 0.47–0.55]; *p* < .001), yogurt (OR = 0.86 [95% CI: 0.79–0.93]; *p* ≤ .001), and coffee (OR = 0.82 [95% CI: 0.76–0.89]; *p* ≤ .001).

**Conclusions:**

The findings of this study indicate a significant relationship between sleep duration and unhealthy food habits. Therefore, increasing awareness of families in this area may reduce obesity and its complications.

## Graphical abstract

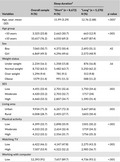

## 1 INTRODUCTION

Obesity in children and adolescents is a major public health problem not only in industrialized countries but also in some developing countries. About 15% of Iranian children are obese and overweight (Kelishadi, 2007). Obesity during adolescence is a predictor of obesity in adulthood (Craigie et al., 2009). The recent epidemic of obesity in children and adolescents has occurred in parallel with the reduction in sleep duration (Dollman, Ridley, Olds, & Lowe, 2007; Iglowstein, Jenni, Molinari, & Largo, 2003; Knutson, 2010). Today, sleep duration in children and adolescents has decreased compared to a decade ago (Matricciani, Olds, & Petkov, 2012). Recently, global review studies have shown a relationship between sleep deprivation and obesity in children and adults (Cappuccio et al., 2008; Chen, Beydoun, & Wang, 2008; Patel & Hu, 2008). In addition, sleep duration is associated with some cardiometabolic risk factors in children and adolescents (Guo et al., 2011). Most eating habits and dietary patterns are formed in childhood and adolescence, and will be consistent throughout the life. These habits are associated with long‐term health, especially with cardiovascular risk factors in adulthood (Anderson, 1991; Kaikkonen et al., 2013).

Snacks are important in providing energy and nutrients, as it is reported that snacks provide about 40% of the daily energy of Iranian students (Dadkhah et al., 2008). It is reported that short sleep is associated with an increase snacking and preferences of high‐energy foods (Chaput, 2014). Mechanism through which inadequate sleeping increases energy intake includes more time and opportunity for eating, psychological distress, more energy need to endure long awakening, and increased appetite hormones (Chaput, 2014).

Limited studies have focused on the relationship between sleep duration and dietary habits, which found different results. A cross‐sectional study by Westerlund showed that less sleep time is associated with increased consumption of high‐energy foods such as fast foods and sweet snacks (Westerlund, Ray, & Roos, 2009). A number of cross‐over clinical trials have shown that limiting sleep causes more tendency for adolescents to eat high‐energy foods such as sweets and desserts (Beebe et al., 2013; Simon, Field, Miller, DiFrancesco, & Beebe, 2015). Cross‐sectional and review studies in adults (Dashti, Scheer, Jacques, Lamon‐Fava, & Ordovas, 2015; Stamatakis & Brownson, 2008) and cohort study in children and cross‐sectional studies in adolescents showed that reducing the duration of sleep is associated with lower intake of fruits and vegetables (Garaulet et al., 2011; Tatone‐Tokuda et al., 2012). A cross‐sectional study on 11‐ to 13‐year‐old Chinese adolescents showed a relationship between sleep deprivation and increased snack consumption only in boys (Lu, Hou, Sun, Zhang, & Tao, 2014).

In a cross‐sectional study on 1,870 students in Massachusetts, it was shown that inadequate sleep duration of less than 10 hr was associated with more drinking of soda and fewer intakes of vegetables, after adjustment for confounders. However, there was no correlation between sleep duration and the consumption of fruits and juices (Franckle et al., 2015).

Considering the increasing prevalence of obesity in children and adolescents, also due to high consumption of high calorie and low nutritive snacks in Iranian children and adolescents (Damari, Riazi‐Isfahani, Hajian, & Rezazadeh, 2015), knowing the relationship between sleep duration and food habits is urgent, to plan and implement effective interventions to prevent obesity and overweight. Therefore, this study aimed to investigate the relationship between sleep duration and snacking in a national sample of children and adolescents aged 6–18 years old.

## 2 METHODS

Data of this study were obtained from the fifth phase of (CASPIAN‐V) study (2014–2015). Details of the procedure, including data collection and the sampling frame, have been described before (Motlagh et al., 2017). In brief, it was a national and cross‐sectional multicenter study on 6‐ to 18‐year‐old students with the cooperation and coordination of the Ministry of Health and Medical Education, Ministry of Education, [Isfahan and Tehran] University of Medical Sciences, and [removed for blind peer review] University of Medical Sciences. The study protocol was reviewed and approved by the Ethics Committee and other related organizations. After explanation of the research goals for students and their parents, oral and written consent was obtained from students and their parents; respectively. Students were selected randomly from cities and villages in a multistage cluster sampling. Forty‐eight clusters of 10 subjects were selected in each province. In total, 14,400 subjects were selected from 30 provinces of the country.

Required data were collected by structured questionnaire, clinical examinations, and anthropometric measurements. The internal consistency of the questionnaire was confirmed in previous studies of CASPIAN with Cronbach's alpha of 0.93 and range of 0.92 to 0.97, and reliability was confirmed by test and retest and correlation coefficient of 0.49 (Azizi‐Soleiman et al., 2016).

Questions which were asked from students included demographic data such as age, gender, place of residence, sleep duration, dietary habits, physical activity, and duration of watching TV and computer work. In addition, a number of questions including the family's social and economic status (parent's education and occupation, having computer, and type of school), and a family history of chronic diseases were asked from parents.

The score of the socioeconomic status was obtained through factor analysis (PCA) and based on data of education and occupation of parents, having computer, type of school (government and private), type of home (personal, rental, and organizational), and having car.

The score was broken down in terms of tertiles: The first tertile was considered as low socioeconomic status, the second tertile as medium level, and the third tertile as high socioeconomic status.

The amount of physical activity was assessed using two questions: 1) "*How many days in the last week you had a 30 min/day physical activity?*" The responses ranged from 0 to 7 days. 2) “*How many hours a week you regularly attend school sports?*” The responses ranged from 0 to ≥ 3 hr.

The duration of watching TV and using computers was also evaluated by the questionnaire. Students reported that they often watch TV and video for hours at their spare time every day.

Responses were in a Likert scale as follow: a) I do not look at all, b) About one hour, c) About 2 hr, d) About 3 hr, and e) About 4 hr or more. Students also reported that they often spend some hours in their free time on working with computers (games, email, chat, or surf and search through the Internet). Responses were similar to the items of watching TV, and for school days and weekends. The average of working computer and watching TV per day was calculated for each student. The duration of watching TV and working with the computer was considered as low (<2 hr a day) and high (>2 hr a day).

In the present study, sleep duration was also evaluated by a questionnaire. Students report that they usually sleep several hours in school days and weekends. The average hours of sleeping per day were calculated and considered as a sleep duration per day for student.

Sleep duration was then considered as a dichotomous variable. The cutoff point of sleeping duration for children < 10 years was 10 hr per day and for children ≥ 10 years was 9 hr per day (Hitze et al., 2009).

To assess nutritional habits of students, 16 food groups were considered including the following: sweets (cakes, muffins, sweets, biscuits, and chocolates), salty snacks (puffs and chips), carbonated beverages, diet soft drinks, soft beer, fresh fruits, dried fruits, fresh juices, vegetables (fresh or cooked), packed juices, milk, yogurt, cheese and fast foods (sausages, hamburgers, and pizzas), tea, sugar along with tea, and coffee. Responses were in a Likert scale as follows: Never (score 1), rarely (score 2), weekly (score 3), and daily (score 4).

Anthropometric measurements included weight, height, and waist circumference according to the standard protocol and conducted by a trained team. Height was measured using a nonelastic tape in a standing position and without shoes, while the scapula was in normal position. Students' scores were measured using a calibrated scale. Waist circumference was measured using a nonelastic meter. Abdominal obesity was considered as waist circumference to height of more than 0.5 (Knowles et al., 2011).

To compare quantitative and qualitative variables, independent *t* test and chi‐square were used; respectively. Quantitative variables were reported as mean and standard deviations, and qualitative variables were reported as frequency and percentage. Then, an ordinal logistic regression was used to determine the relationship between sleep duration and dietary habits. Sleep duration was considered as an independent variable as a dichotomous variables (cutoff point for sleep duration was 10 hr a day for children < 10 years and 9 hr for children and adolescents ≥ 10 years). Each food item was entered the model as a dependent variable. Probable confounders including age, sex, place of residence, BMI, socioeconomic status, physical activity, duration of watching TV and computer work, parents’ BMI, history of chronic diseases (blood pressure, blood lipids, diabetes, and obesity), and also two psychological problems (1—Anxiety over the past 6 months, 2—“During the past 12 months, did you ever feel so sad or hopeless for two weeks or more that you stopped doing some usual activities?”) were entered a multivariate logistic regression model. In addition, another analysis was performed and the sleep duration was entered to the multivariate logistic regression model as a quantitative variable. The data were analyzed using STATA 10 software (Stata Corp). The significance level was considered 0.05.

## 3 RESULTS

Out of 14,274 students, 7,228 (50.7%) were boys. 71.4% of the students were urban residents. The mean (*SD*) age of the participants was 12.28 (3.16) years. The average (*SD*) of students' sleep duration was 8.57 (1.23) hours per day.

The characteristics of students in terms of sleep duration are presented in Table 1. There was a significant relationship between age, socioeconomic status, place of residence, physical activity, and duration of watching TV and working with computer with sleep duration (*p* < .001). Urban students had lower physical activity, longer computer work, higher socioeconomic status, and shorter sleep duration (Table 1). According to the chi‐square test, there was a significant correlation between all food groups (except the group tea with sugar) and sleep duration (Table 2).

**Table 1:** Characteristics of the study subjects according to daily sleep duration

Variables	Overall sample *N* (%)	Sleep duration^a^		*p*‐value
		“Short” (*n* = 8,672) *N* (%)	“Long” (*n* = 5,270) *N* (%)	
Age, year; mean (*SD*)		11.99 (3.29)	12.76 (2.88)	<.001*
Age group				
<10 years	3,325 (23.8)	2,662 (30.7)	663 (12.9)	<.001
≥10 years	10,617 (76.2)	6,010 (69.3)	4,607 (87.4)	
Sex				
Boy	7,065 (50.7)	4,372 (50.4)	2,693 (51.1)	.42
Girl	6,869 (49.3)	4,296 (49.6)	2,573 (48.9)	
Weight status				
Under weight	2,234 (16.1)	1,358 (15.8)	876 (16.8)	.16
Normal weight	8,732 (63.1)	5,482 (63.7)	3,250 (62.2)	
Over weight	1,294 (9.4)	781 (9.1)	513 (9.8)	
Obese	1579 (11.4)	991 (11.5)	588 (11.2)	
SES				
Low	4,451 (33.4)	2,701 (32.6)	1,750 (34.6)	.001
Moderate	4,420 (33.2)	2,703 (32.7)	1717 (34)	
High	4,460 (33.5)	2,807 (34.7)	1,590 (31.4)	
Living area				
Urban	9,934 (71.3)	6,267 (72.3)	3,667 (69.6)	.001
Rural	4,008 (28.7)	2,405 (27.7)	1603 (30.4)	
Physical activity				
Low	4,399 (33.7)	2,898 (35.9)	1501 (30.2)	<.001
Moderate	4,333 (33.2)	2,614 (32.4)	1719 (34.5)	
High	4,312 (33.1)	2,556 (31.7)	1756 (35.3)	
Watching TV				
Low	6,422 (46.1)	4,147 (47.8)	2,275 (43.3)	<.001
High	7,507 (53.9)	4,522 (52.2)	2,985 (56.7)	
Working with computer				
Low	12,393 (91)	7,657 (89.7)	4,736 (93.1)	<.001

Abbreviations: BMI, body mass index; SES, socioeconomic status; *SD*, standard deviation.

a Short sleep duration: 10 hr per day for children aged < 10 years and 9 hr per day for children/adolescents aged ≥ 10 years.

* According to the *t* test, other *p*‐values are based on chi‐square test.

**Table 2:** Association of the frequency of snack consumption with sleep duration: The CASPIAN‐V study

Snack type	Short sleep duration a		*p*‐value*
	Yes (*n* = 8,672)	No (*n* = 5,270)	
Sweets			
Never	161 (1.9)	212 (4)	<.001
Rarely	2,825 (32.6)	1993 (37.8)	
Weekly	3,849 (44.4)	1994 (37.8)	
Daily	1836 (21.2)	1,071 (20.3)	
Salty snacks			
Never	1,068 (12.3)	1,061 (20.3)	<.001
Rarely	4,537 (52.5)	2,615 (50)	
Weekly	2,429 (28.1)	1,419 (27.1)	
Daily	615 (7.1)	138 (2.6)	
Soft drinks			
Never	2,119 (24.5)	1,457 (27.7)	<.001
Rarely	4,117 (47.5)	2,287 (43.4)	
Weekly	2,165 (25)	1,333 (25.3)	
Daily	265 (3.1)	187 (3.6)	
Diet soda			
Never	6,738 (78.1)	3,923 (75.1)	<.001
Rarely	1585 (18.4)	926 (17.7)	
Weekly	223 (2.6)	361 (6.9)	
Daily	82 (1)	16 (0.3)	
Delester			
Never	3,818 (44.6)	2,197 (42.8)	.013
Rarely	3,172 (37)	2028 (39.5)	
Weekly	1,184 (13.8)	706 (13.8)	
Daily	392 (4.6)	201 (3.9)	
Fresh fruits			
Never	243 (3.2)	183 (4)	.001
Rarely	656 (8.6)	439 (9.6)	
Weekly	2,196 (28.9)	1,201 (26.1)	
Daily	4,515 (59.3)	2,770 (60.3)	
Dried fruits			
Never	457 (5.5)	357 (7.4)	<.001
Rarely	2,258 (27.2)	517 (10.7)	
Weekly	3,314 (39.9)	1773 (36.7)	
Daily	2,285 (27.5)	2,190 (45.3)	
Fresh juices			
Never	707 (8.2)	355 (6.8)	<.001
Rarely	3,866 (44.8)	1729 (33.1)	
Weekly	2,873 (33.3)	1941 (37.2)	
Daily	1,188 (13.8)	1,198 (22.9)	
Canned fruit juice			
Never	1,177 (13.8)	606 (11.5)	<.001
Rarely	3,577 (41.9)	2,315 (44)	
Weekly	2,867 (33.6)	1731 (32.9)	
Daily	913 (10.7)	608 (11.6)	
Vegetables			
Never	474 (5.5)	373 (7.1)	<.001
Rarely	1,112 (13)	853 (16.3)	
Weekly	4,237 (49.5)	2,345 (44.9)	
Daily	2,734 (32)	1653 (31.6)	
Milk			
Never	679 (7.8)	141 (2.7)	<.001
Rarely	1,195 (13.8)	304 (5.8)	
Weekly	3,685 (42.5)	2,260 (42.9)	
Daily	3,112 (35.9)	2,561 (48.6)	
Yogurt			
Never	264 (3.1)	28 (0.5)	<.001
Rarely	1,104 (12.9)	460 (8.7)	
Weekly	2,499 (29.1)	1655 (31.4)	
Daily	4,709 (54.9)	3,121 (59.3)	
Cheese			
Never	411 (4.7)	358 (6.8)	<.001
Rarely	618 (7.1)	291 (5.5)	
Weekly	1583 (18.3)	959 (18.2)	
Daily	6,055 (69.9)	3,656 (69.5)	
Fast foods			
Never	1,397 (16.1)	1,236 (23.5)	<.001
Rarely	4,721 (54.5)	2,821 (53.5)	
Weekly	1,468 (16.9)	707 (13.4)	
Daily	1,079 (12.5)	504 (9.6)	
Tea			
Never	948 (11)	869 (16.5)	<.001
Rarely	1,009 (11.7)	750 (14.3)	
Weekly	1995 (23.1)	1,200 (22.8)	
Daily	4,694 (54.3)	2,436 (46.4)	
Sugar			
Never	860 (9.9)	569 (11)	.07
Rarely	1733 (20)	986 (19)	
Weekly	1814 (21)	1,132 (21.8)	
Daily	4,250 (49.1)	2,496 (48.2)	
Coffee			
Never	3,700 (42.7)	2,533 (48.1)	<.001
Rarely	3,886 (44.9)	1,429 (27.1)	
Weekly	718 (8.3)	883 (16.8)	
Daily	360 (4.2)	423 (8)	

a Short sleep duration: 10 hr per day for children aged < 10 years and 9 hr per day for children/adolescents aged ≥ 10 years.

* Comparisons based on chi‐square test.

To assess the relationship between sleep duration and dietary habits of students, an ordinal logistic regression model was used and probable confounders were adjusted. Sleep duration was entered multivariate regression model as a dichotomous and qualitative independent variable. Short sleep compared to long sleep was associated with increased chance of consuming salty snacks, carbonated drinks, fast foods, tea, and tea with sugar (Table 3). In addition, short sleep in students is associated with lower odds of daily intake of diet soft drinks, soft beer, fresh fruits, dried fruits, fresh fruit juices, packed juices, milk, yogurt, and coffee (Table 3).

**Table 3:** Association of sleep duration with frequency of snacks consumption in ordinal logistic regression model: the CASPIAN‐V study

		Sweets	Salty snacks	Soft drinks	Diet soda	Delester	Fresh fruits	Dried fruits	Fresh juices	Canned fruit juice	Vegetables	Milk	Yogurt	Cheese	Fast foods	Tea	Sugar	Coffee
Short sleep a	OR (95% CI)^b^	1.28 (1.19–1.36)	1.49 (1.39–1.59)	1.06 (0.99–1.13)	0.82 (0.75–0.88)	0.97 (0.91–1.03)	0.99 (0.93–1.07)	0.48 (0.45–0.51)	0.59 (0.56–0.63)	0.94 (0.88–1.003)	1.14 (1.07–1.2)	0.52 (0.49–0.55)	0.77 (0.72–0.82)	1.03 (0.96–1.11)	1.47 (1.38–1.58)	1.44 (1.35–1.53)	1.03 (0.97–1.10)	0.92 (0.86–0.98)
	*p*‐Value b	<.001	<.001	.09	<.001	.32	.89	<.001	<.001	.06	<.001	<.001	<.001	.38	<.001	<.001	.29	.01
	OR (95% CI)^c^	1.05 (0.98–1.13)	1.49 (1.38–1.61)	1.12 (1.04–1.20)	0.64 (0.58–0.70)	0.92 (0.85–0.99)	0.83 (0.76–0.90)	0.43 (0.39–0.46)	0.66 (0.62–0.72)	0.91 (0.84–0.98)	1.02 (0.95–1.10)	0.51 (0.47–0.55)	0.86 (0.79–0.93)	1.02 (0.93–1.10)	1.66 (1.54–1.79)	1.49 (1.39–1.61)	1.13 (1.05–1.22)	0.82 (0.76–0.89)
	*p*‐Value c	.19	<.001	.002	<.001	.02	<.001	<.001	<.001	.009	.56	<.001	<.001	.71	<.001	<.001	.001	<.001
Sleep duration^a^	OR (95% CI)^b^	0.99 (0.97–1.02)	0.92 (0.89–0.94)	0.99 (0.96–1.01)	1.1 (1.08–1.15)	1.04 (1.01–1.06)	1.01 (0.98–1.04)	1.40 (1.36–1.44)	1.30 (1.27–1.34)	1.09 (1.07–1.12)	0.96 (0.93–0.98)	1.49 (1.45–1.53)	1.18 (1.15–1.21)	0.94 (0.91–0.96)	0.91 (0.89–0.93)	0.77 (0.75–0.79)	0.93 (0.90–0.95)	1.11 (1.08–1.14)
	*p*‐Value b	.76	<.001	.29	<.001	.007	.43	<.001	<.001	<.001	.001	<.001	<.001	<.001	<.001	<.001	<.001	<.001
	OR (95% CI)^c^	1.1 (1.08–1.14)	1 (0.98–1.04)	1 (0.97–1.03)	1.22 (1.18–1.27)	1.12 (1.09–1.16)	1.08 (1.04–1.11)	1.45 (1.4–1.49)	1.26 (1.23–1.3)	1.08 (1.05–1.12)	1.0 (0.98–1.04)	1.58 (1.53–1.63)	1.12 (1.08–1.15)	0.92 (0.89–0.95)	0.88 (0.85–0.90)	0.79 (0.77–0.82)	0.89 (0.86–0.92)	1.20 (1.16–1.24)
	*p*‐Value^c^	<.001	.73	.98	<.001	<.001	<.001	<.001	<.001	<.001	.43	<.001	<.001	<.001	<.001	<.001	<.001	<.001

a Short sleep duration: 10 hr per day for children aged < 10 years and 9 hr per day for children/adolescents aged ≥ 10 years.

b Without adjusted (crude model).

c Adjusted for age, sex, living area, socioeconomic status, physical activity, BMI, screen time**,** parental obesity, family history of chronic diseases, anxiety, and depression

In another analysis, sleep duration was entered the model as a qualitative variable. Results showed that higher sleep duration is associated with increased odds of daily intake of cakes, diet drinks, soft beer, fresh fruits, dried fruits, fresh juices, packed juices, milk, yogurt, and coffee. The findings of this study also showed that with every one hour increase in sleep duration, the odds of daily intake of cheese, fast food, tea, and sugar decreased. However, there was no significant relationship between sleep duration and consumption of salty snacks, carbonated soft drinks, and vegetables (Table 3).

## 4 DISCUSSION

The results of this study showed that low sleep duration compared to long sleep is significantly associated with increased odds of daily intake of salty snacks, soft drinks, fast foods, tea, and tea with sugar. In addition, low sleep duration in students is associated with lower odds of daily consumption of diet drinks, soft beer, fresh fruits, and dried fruits, fresh juices, packed juices, milk, yogurt, and coffee. However, there was no significant relationship between sleep duration and consumption of cake, cheese, and vegetables.

Results of studies which assessed the relationship between sleep duration and unhealthy eating habits in children and adolescents are consistent with the current study. Results from a recent meta‐analysis also supported that short sleep duration was specifically related to more consumption of soda, snacks, and fewer fruits in children and adolescents (Cordova, Barja, & Brockmann, 2018).

Chaput et al. in a multinational cross‐sectional study on 5,777 children aged 9–11 years showed that lower sleep duration and later bedtime are significantly associated with unhealthy food pattern (Chaput et al., 2015). A cross‐sectional study on 1,265 students aged 9–11 years in Finland showed that there is a relationship between inadequate sleep and unhealthy energy‐rich food patterns including the following: pizza, hamburger, hot dogs or meat pastry; crisps or popcorn; cookies; ice cream; sweets or chocolate; and coca cola or other soft drinks (Westerlund et al., 2009).

A cross‐sectional study on 3,311 European adolescents showed that frequency of eating junk foods such as pizza, hamburger, and pasta snack products is higher in people who have a short sleep duration (Garaulet et al., 2011). A cohort study (2012) in Canada showed that short sleep times are associated with increased consumption of meat and meat products/alternatives in boys and an increase in the consumption of soft drinks in girls (Tatone‐Tokuda et al., 2012).

A cohort study in Canada showed that short sleep duration is associated with increased consumption of meat and meat products/alternatives in boys and increased consumption of soft drinks in girls (Tatone‐Tokuda et al., 2012). Franckle et al. (2015) also showed that sleep duration less than 10 hr is associated with more drinking of soda. A prospective study in Denmark on children aged 8–11 years showed that lower sleep duration is associated with higher intake of sugar containing foods/beverages (Hjorth et al., 2014). In an experimental study, sleep deprivation was associated with increased appetite for high calorie and high carbohydrate foods such as sweets, salty snacks (chips, salted nuts, pickles, and olives), and starchy foods (bread, pasta, cereal, and potatoes) (Spiegel, Tasali, Penev, & Van Cauter, 2004).

Some studies have also examined the relation between sleep duration and the amount of energy derived from carbohydrate, protein, and fat. Martinez et al. in a cross‐sectional study showed that longer sleep duration is associated with lower carbohydrate intake (Martinez et al., 2017).

Weiss et al. (2010) in a cross‐sectional study in adolescents showed that less sleep duration is associated with increased odds of consuming calories from fat (Weiss et al., 2010). An experimental study on 37 children aged 8–11 years showed that higher sleep duration in students was associated with an average of 134 kcal/day less and lower weight (Hart et al., 2013).

In addition, the findings of this study showed that short sleep duration in students is associated with lower odds of daily intake of fresh fruits, dried fruits, fresh fruit juice, milk, yogurt, and coffee. There are some similar studies in adults. A review study showed that short sleep duration is associated with fewer fruits and vegetables intake and consumption of low‐quality foods (Dashti et al., 2015). A cross‐sectional study by Stamatakis and Brownson (2008) on 1,203 adults showed that short sleep has a significant relationship with obesity‐related behaviors, in particular with lower physical activity and reduced consumption of fruits and vegetables. A cross‐sectional study on 410 female students aged 18–28 years in Isfahan showed that overweight and obesity is more prevalent among those who sleep <6 hr per day. In addition, energy and carbohydrate intake were higher, while protein, fiber, fruits, whole grains, and beans intake were lower (Haghighatdoost, Karimi, Esmaillzadeh, & Azadbakht, 2012).

There are also some similar studies in children. A cohort study in Canada showed that short sleep duration in children was associated with lower consumption of fruits and vegetables in both genders and lower dairy consumption in girls (Tatone‐Tokuda et al., 2012). A cross‐sectional study on 3,311 European adolescents showed that shorter sleep duration is associated with fewer fruits and vegetables consumption (Garaulet et al., 2011). While Westerlund et al. (2009) showed a relationship between short sleep duration and lower consumption of healthy foods such as fruits, vegetables, and nutrient‐dense foods only in girls, a recent cross‐sectional study on 1,870 students in Massachusetts showed that after adjustment for confounding factors, inadequate sleep duration of less than 10 hr was associated with lower consumption of vegetables. However, there was no relationship between sleep duration and consumption of fruit and juice drinks (Franckle et al., 2015).

In the present study, no relationship was found between sleep duration and vegetables intake. Similar results were shown in a study on Chinese adults (Tu et al., 2012).

However, a systematic review (2018) focusing on the pediatric population showed that low sleep duration was associated with fewer vegetables and fruits (Cordova et al., 2018).

A cross‐sectional study showed that sleeping less during school nights is associated with lower consumption of nutrient‐dense foods, such as fruits and vegetables (Westerlund et al., 2009).

The reasons for the inconsistent findings may be the difference in characteristics of participant, methods of dietary intakes assessment, and different cultural conditions.

It could be due to the nutritional culture of the people, so that consuming more vegetables with main meals is less affected by reduced sleep duration. In an experimental study, it was shown that sleep deprivation (in an obesity‐promoting environment) is accompanied by increased intake of calorie from snacks, but without any significant changes in calorie intakes from main meals (Nedeltcheva et al., 2009).

Sleep restriction may affect an individual's food intake by altering circadian cycle and by altering the level of hunger and satiety hormones (leptin and ghrelin), as well as by altering blood glucose levels (Knutson, Spiegel, Penev, & Van Cauter, 2007; Magee, Huang, Iverson, & Caputi, 2010; Spiegel, Tasali, Leproult, & Van Cauter, 2009). It seems that sleep deprivation results in increased food intake through increasing appetite‐stimulating hormone (ghrelin) and decreasing appetite‐suppressing hormone (leptin) (Spiegel et al., 2004; Zadeh & Begum, 2011). In addition, individuals with sleep deprivation have more time to eat (Chaput et al., 2010; Qin et al., 2003). On the other hand, short sleep may lead to disordered consumption of the main meals and increased intake of junk foods and unhealthy snacks (Dashti et al., 2015).

It seems that short sleep duration increases the prevalence of obesity through changing hormone levels and probably by increasing the exposure of children to certain environmental factors such as watching TV (Garaulet et al., 2011; Magee, Caputi, & Iverson, 2014). As a study in children and adolescents showed, most children and adolescents watch TV before bedtime and screen time before sleeping delays bedtime (Foley et al., 2013).

Chaput et al. in a multinational cross‐sectional study on 5,777 children aged 9–11 years showed that short sleep duration is associated with more sedentary behaviors. Similarly, later bedtimes are associated with an increase in sedentary behaviors and screen time (Chaput et al., 2015). Sedentary behaviors (like watching TV) are also associated with unhealthy eating habits such as increased intake of high‐energy snacks and fast foods and increased energy intake and reduced consumption of fruits and vegetables in children and adolescents (Azizi‐Soleiman et al., 2016; Pearson & Biddle, 2011; Safiri et al., 2016). So, it increases overweight and obesity. As a review study shows, there is a positive correlation between unhealthy food patterns and cardiometabolic risk factors in children and adolescents (Rocha, Milagres, Longo, Ribeiro, & Novaes, 2017).

Some factors make comparing the findings of various studies difficult, including method of dietary intakes assessment (food frequency questionnaire, 24‐hr food recall), different definitions of sleep deprivation, different age groups, differences in analysis method, and difference in adjusted confounding factors.

In addition, food habits vary in different societies and may be influenced by the specific factors of that community, including the cultural level and the customs and customs of the community, attitude, role of parents, environmental conditions, food variety, and the extent of food access, as well as the modeling of peers.

Some limitations are outlined. First, it was a cross‐sectional study and a causal inference cannot be established. Other limitations include the method of collecting lifestyle information (dietary habits, physical activity, sedentary behaviors, and sleep duration) which was based on a self‐administered questionnaire, and thus, recall bias is probable.

In addition, if food items were collected quantitatively, intakes were determined more precisely. Also, despite the adjustment for some confounding variables, there were no data on puberty status and household income. As a result, they may affect the outcome of the study. In addition, there may be unknown confounding factors that are not controlled in this study.

The age range of participants in the current study was 6–18 years old. A study showed that sleep duration was associated with age, and older children suffer from greater sleep deprivation (Hitze et al., 2009). Sleep duration in children and adolescents may also be affected by factors such as puberty, physiological changes, and psychology.

On the other hand, the extent of parental supervision on food consumption may be related to the age of student. So that children are more likely to be supervised by parents than adolescents. Adolescents have more freedom to spend their leisure time outside the home compared to children, and they may be influenced by their peers in choosing snacks.

Large sample size was one of the strengths of this study. Second, the WHO standard questionnaire was used in this study. In addition, a large number of possible confounding factors were controlled in this study. Finally, high‐quality data gathering was another strength of the present study.

## 5 CONCLUSIONS

In conclusion, sleep duration can be associated with some unhealthy eating habits and as a result with obesity in children and adolescents. Therefore, it is possible to prevent obesity and its complications by increasing the awareness of families in this area and controlling their sleep duration and their eating habits. In this study, cross‐sectional relationship between food habits and sleep duration was observed. Prospective and interventional studies in this field are warranted.

## CONFLICT OF INTEREST

The authors declare that they have no competing interests.

## ETHICAL APPROVAL

This study was approved by the Research and Ethics Council of Isfahan University of Medical. Sciences (code: 194,049).

## CONSENT FOR PUBLICATION

Not applicable.

## INFORMED CONSENT

Written informed consent was obtained from all study participants.

## Data Availability

The datasets used and analyzed during the current study are available from the corresponding author on reasonable request.
